# Evaluating time between birth to cry or bag and mask ventilation using mobile delivery room timers in India: the NICHD Global Network’s Helping Babies Breathe Trial

**DOI:** 10.1186/s12887-015-0408-6

**Published:** 2015-08-06

**Authors:** Manjunath S. Somannavar, Shivaprasad S. Goudar, Amit P. Revankar, Janet L. Moore, Elizabeth M. McClure, Pablo Destefanis, Martha DeCain, Norman Goco, Linda L. Wright

**Affiliations:** KLE University’s Jawaharlal Nehru Medical College, Belgaum, Karnataka India; RTI International, Durham, NC USA; Eunice Kennedy Shriver National Institute of Child Health and Human Development, Bethesda, MD USA

**Keywords:** Resuscitation, Golden minute, Asphyxia, India, Helping babies breathe

## Abstract

**Background:**

The Golden Minute®, the first minute following birth of a newborn, is a critical period for establishing ventilation after delivery, as emphasized in the Helping Babies Breathe® and other resuscitation training programs. Previous studies have reinforced training through observers’ evaluation of this time period; although observation is useful for research, it may not be a sustainable method to support resuscitation practice in low-resource settings where few birth attendants are available. In order to reinforce resuscitation within The Golden Minute®, we sought to develop a simple mobile delivery-room timer on an Android cell phone platform for birth attendants to use at the time of delivery.

**Methods:**

We developed and evaluated a mobile delivery room timer to document the time interval from birth to the initiation of newborn crying/spontaneous respiration or bag and mask ventilation in a convenience sample of women who delivered in five hospitals in Karnataka, India. The mobile delivery room timer is an Android cell phone-based application that recorded key events including crowning, delivery, and crying/spontaneous respiration or bag and mask ventilation. The mobile delivery room timer recorded the birth attendant verbally indicating the time of crowning, birth-(defined as when the entire baby was delivered), crying/spontaneous respiration or bag and mask ventilation. The mobile delivery room timer results were validated in a subsample by a trained observer (nurse) who independently recorded the time between delivery and initiation of crying/spontaneous respiration or bag and mask ventilation.

**Results:**

Of the total 4,597 deliveries, 2,107 (46 %) were timed; a sample (*n* = 438) of these deliveries was also observed by a trained nurse. There was high concordance between the mobile delivery room timer and observed time elapsed between birth and crying/spontaneous respiration or ventilation (correlation =0.94, *p* < 0.0001). The majority of neonates in both groups cried/breathed spontaneously or received bag and mask ventilation by 1 min (430/438 by the timer vs. 433/438 for observer).

**Conclusions:**

We demonstrated that a simple mobile delivery room timer application was feasible to use during delivery and provided valid observations of the time to crying/spontaneous respiration or bag and mask ventilation. This type of tool may be useful in reinforcing neonatal resuscitation training and the need to ensure spontaneous or assisted ventilation by The Golden Minute®.

## Background

Helping Babies Breathe® (HBB), a simulation-based curriculum designed to teach resuscitation of babies wherever they are born, was developed by the American Academy of Pediatrics (AAP) and partners; it was launched in 2010 [1, 2]. The HBB program targets low and middle-income country birth attendants (BA) with a combination of evidence-based best practices and graphic action plans and teaching techniques. HBB focuses on providing resuscitation to babies who do not begin to breathe on their own using stimulation (rubbing the back) or stimulation and bag and mask ventilation within the first minute after birth (The Golden Minute®) [2–4]. HBB is focused on essential resuscitation knowledge and skills to help ensure that the BAs become more proficient in neonatal resuscitation.

The *Eunice Kennedy Shriver* National Institute of Child Health and Human Development (NICHD)’s Global Network for Women’s and Children’s Health Research (Global Network) designed the study “*Evaluation of HELPING BABIE*S *BREATHE: Does Implementation of Helping Babies Breathe Save Lives?”* to test the effectiveness of HBB training in low-resource, facility settings with limited exposure to formal resuscitation training [5]. Previous studies have used observers to verify that BAs followed the HBB Action Plan and were able to achieve spontaneous respiration or were ventilated with a bag and mask (BMV) by The Golden Minute®; [2–4, 6, 7] however, use of observers may not be sustainable in low-resource settings where only one BA may be available to attend the birth and resuscitate the newborn [8–10]. This study was conducted after the initial Global Network HBB study training and was designed to develop and validate a mobile delivery-room timer (MDT) to document, evaluate, and reinforce the BA adherence of immediate drying of all newborns, followed by back rubbing/stimulation for those neonates that do not breathe spontaneously, and by resuscitation with BMV to ensure that ventilation occurs within a one minute interval following birth. Our primary hypothesis was that the MDT was feasible to use to accurately document the steps in the resuscitation of vaginal deliveries. The secondary hypothesis were that the MDT would facilitate collection of DR activities and facilitate evaluation of the recommended steps in HBB resuscitation.

## Methods

This study had two components. First, we developed the MDT software and design as an iterative process with the investigators and software developer and then piloting the device in the field team. After the development of the device was completed, we next trained the birth attendants and then conducted a field study in several hospitals which were participating in the HBB trial.

### Development of MDT

A software program was developed to collect and record the data on an Android device. The Android operating system was selected because it is used on a wide range of cell phones and tablets, including phones in the lower end of the cost range. Selection of the Android-based system was important since the project was targeted for use in low-income country settings and thus availability of software which functioned on low-cost hardware was preferred for sustainability. After the software was programmed, it was tested for reliability and usability by RTI staff and further refined before it was deployed and field tested in Belgaum prior to initiation of the validation study.

This software was enabled with an audible mark and a physical mark entry-type system. The physical marks included easy-to-read symbols to indicate, crowning, birth, cry, or BMV. This application was published in Google Play, is available on the Google Play website under the term “Helping Babies Breathe” and may be installed through Google Play at no cost [11].

Development and beta testing of the application was started in October 2012; a stable, ready-to-use version was available in March 2013.

#### MDT

**Fig. 1:**
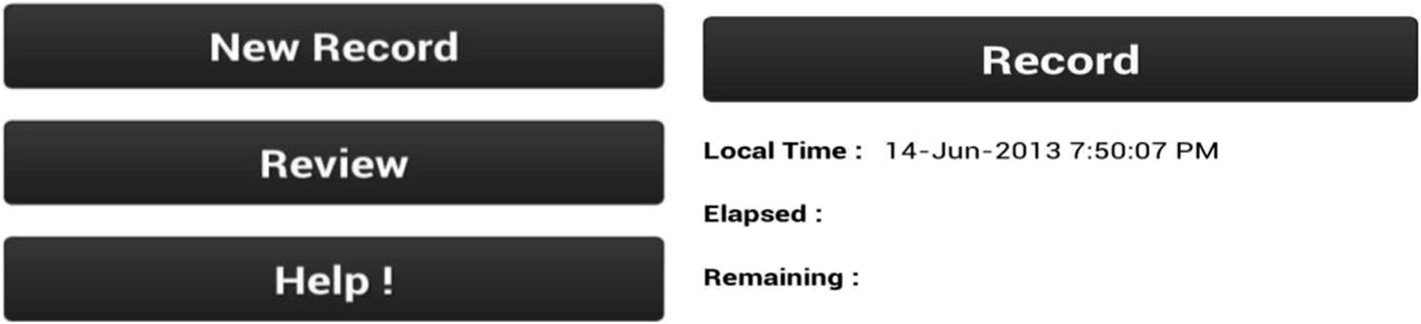
Starting the recording; User Selects “New Record” to start the process and then clicks “Record”

**Fig. 2:**
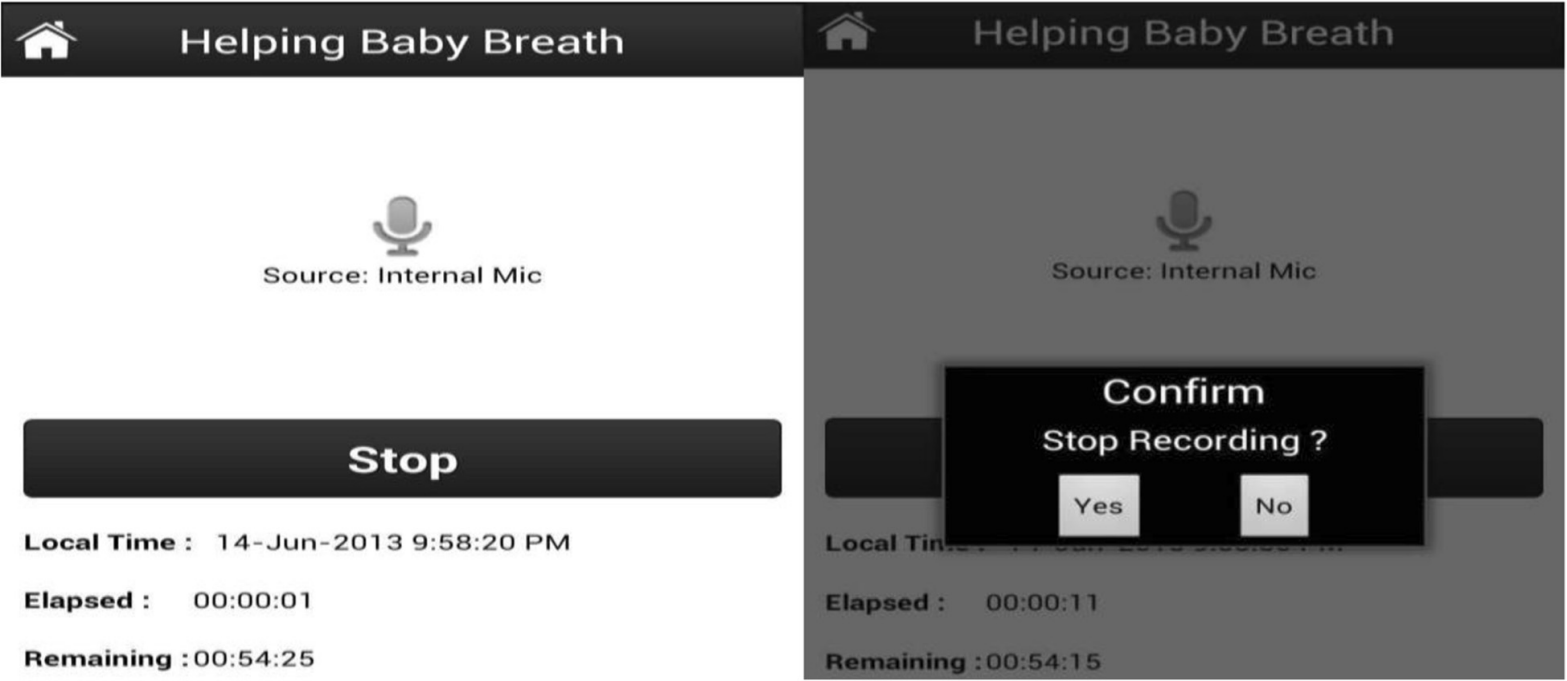
Stopping the recording; When the session is complete, user clicks “Stop” and the system asks for confirmation to end recording

**Fig. 3:**
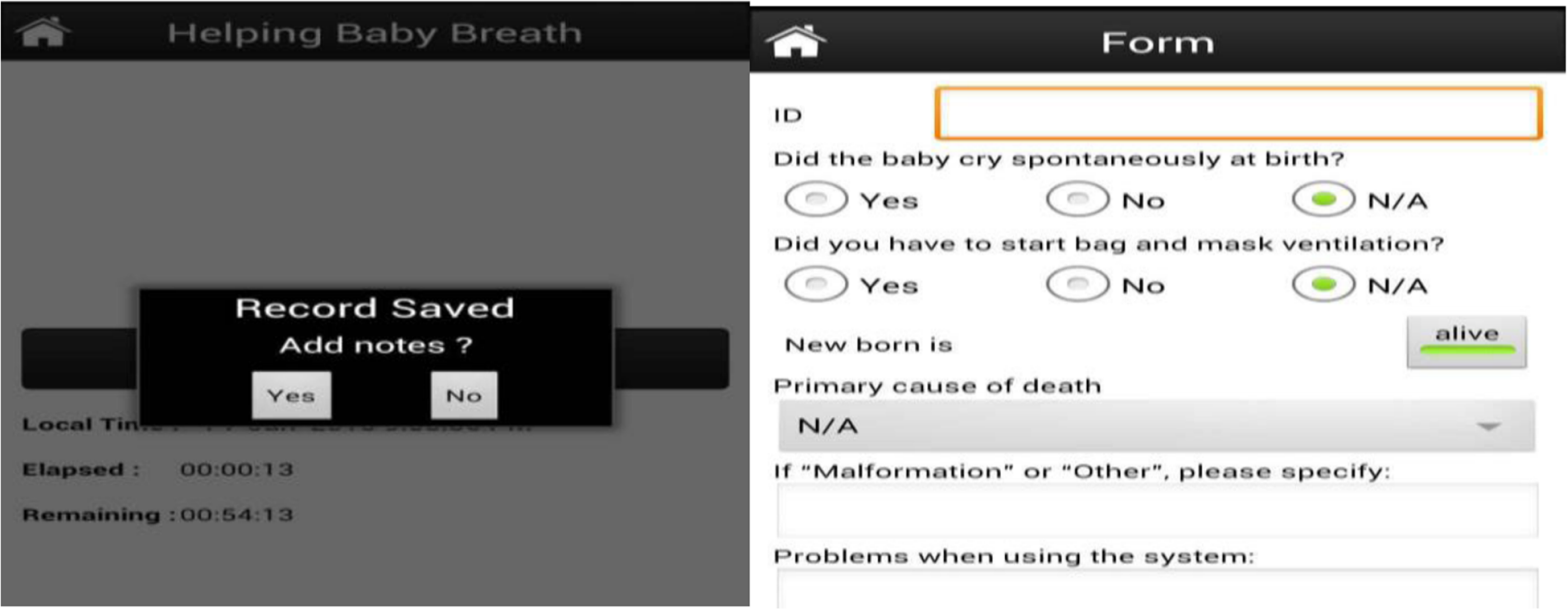
Entering birth outcome information; BA is prompted to enter birth outcomes on the mobile device

To operate the MDT, when a delivery was imminent, the application was turned ‘ON’ (Fig. 1) to begin recording and remained on for the duration of the delivery. Four key points were verbally marked in the recording during the delivery process by the birth attendant who called out: START (when the baby’s head crowned); BIRTH (when the baby was born), and CRY, (if the baby spontaneously cried or took a breath*) or* BAG (if baby did not breathe spontaneously) and the birth attendant used BMV to resuscitate the infant. After the birth activities were completed, the MDT was stopped as illustrated in Fig. 2 and the BA completed data collection by filling in the identification numbers for the birth attendant and participant on the mobile phone (Fig. 3). At a later time, the Facility Coordinator reviewed the recoding and placed the physical mark entry onto the recording.

The device was piloted in the month of April 2013 using a convenience sample of birth attendants who had been trained in HBB.

### Field study

#### Setting

This study was conducted at five of the 32 Belgaum, India health facilities where BAs had been trained in HBB. Three county hospitals, one private hospital and one community health center (CHC) were selected to ensure a sample of at least 100 births per month within the study catchment area. County hospitals had 200–350 births per month, and each of the private hospitals and CHC had 200 and 100 births per month, respectively. Five nurses, one from each facility, were selected as Facility Coordinators to supervise activities at each of these facilities.

#### Training

Birth attendants at these facilities received a three-hour basic training in the use of the MDT device, including the accurate timing of the four key events: Crowning, Birth, Spontaneous Crying/Respiration or BMV. The BA then entered the participant identification number and basic delivery outcome information on the mobile phone. During the delivery, the BA spoke the phrases “Start (when the baby crowned),” “Birth” “Cry” and “Bag” to indicate when these events occurred, which were recorded on the MDT. After delivery, the BA reviewed the recorded information with the Facility Coordinators to ensure that the time was correctly marked on the MDT, as described below. Short paper forms were available to record the information in the event that the timer device was inadvertently not started and the reasons why the MDT was not used.

The BAs also received weekly supervised visits and onsite refresher training to review the enrollment status and use of the devices. The Facility Coordinators were instructed to observe a sample of deliveries (10 %) for which the timer was used. These Facility Coordinators were trained to independently observe births in order to collect the time interval data using a stop watch, i.e., Birth to either cry/spontaneous respiration or initiation of BMV (observer data), independent of MDT data. Facility coordinators were also trained in the process for transferring MDT data to the server.

### Data collection

Data were collected both on the MDT and hard copy. Android cell phones with the MDT and charging units were placed in the delivery rooms of the participating facilities for the BAs to use. A second MDT device was available in each delivery room to record deliveries as a back-up.

**Fig. 4:**
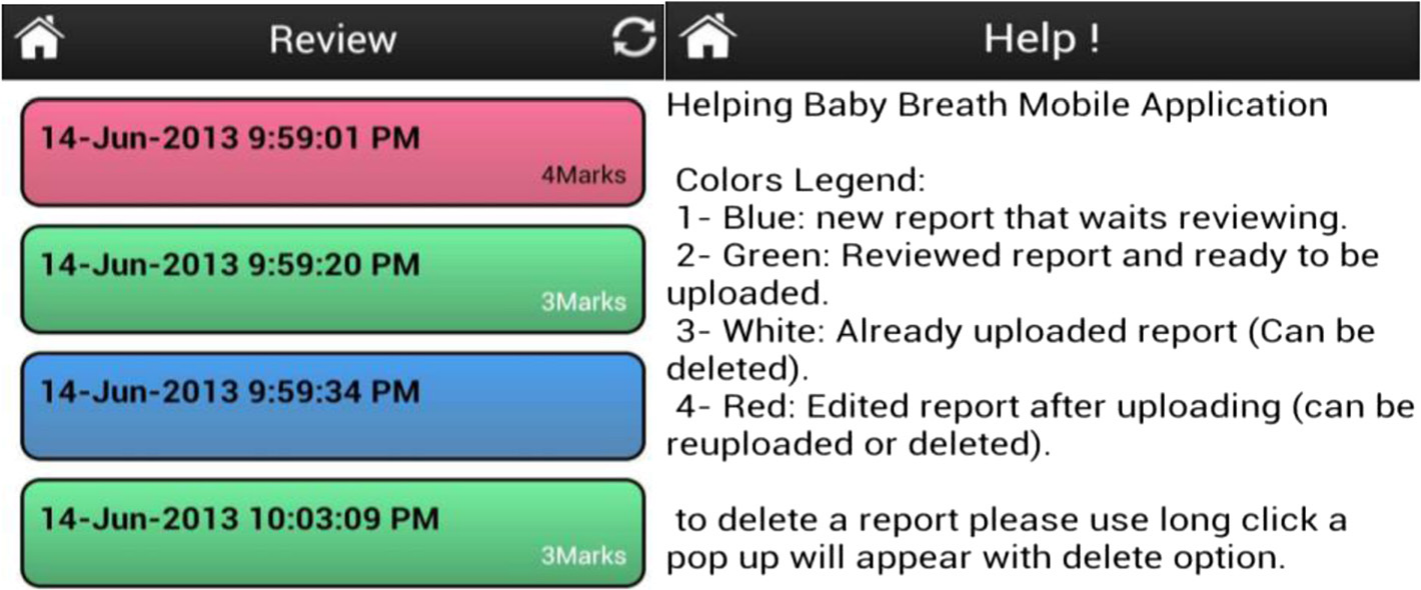
Post-delivery record review; When “Review” is selected, the system shows the records stored in the phone. Blue indicates not reviewed; Green indicates reviewed with an indication of the number of marks entered. The user then selects a session for review and while the recording is being reviewed, is presented with a progress bar, similar to what is seen for an audio recording, and four buttons that allows for placing a timestamp on the recording for the audible cues of “Start/Crown”, “Birth”, “Cry” or “Bag/Resuscitation” as illustrated in Fig. 5

**Fig. 5:**
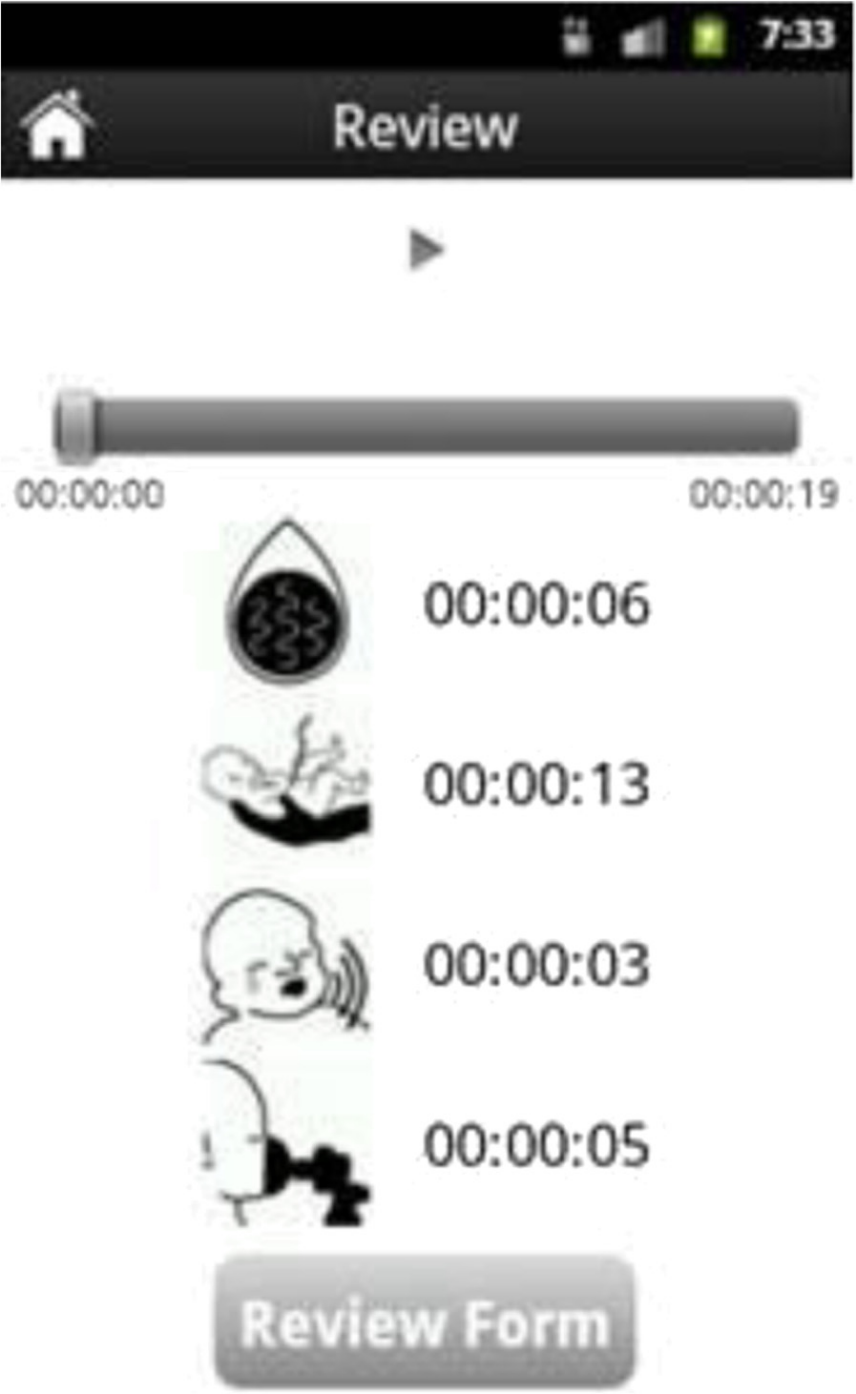
Illustration of progress bar and timestamp mark symbols

The data collected on the MDT device were reviewed by the Facility Coordinator with the BAs (Fig. 4) to ensure that the corresponding markings (i.e., time of crowning, birth, cry or BMV) were completed correctly (Fig. 5). Reviewed records were then uploaded into the Google’s AppEngine service at http://helpingbabybreath.appspot.com on weekly basis. Finally, observers’ log of the events, which were collected for a sub-sample of the deliveries, were collected on paper forms and entered into a separate (Microsoft Access) database by site data entry staff. In addition, paper data collection forms were available for BAs to record the reason for not starting the device if the MDT was not activated.

At the completion of the MDT data collection, a brief survey of the BA’s was conducted in order to assess the MDT’s usefulness to the BAs and improve future design of the device. The survey addressed aspects of training, ease of use of the MDT, capturing data on Android platform and the use of feedback for improving future resuscitation.

### Data management and statistical analysis

Data entry and transmission to the data coordinating center was completed on a weekly basis by trained data entry staff. Edits were performed centrally and resolved at the site to ensure internal consistency of data.

The research team reviewed and corrected data edits centrally on monthly basis. The data were analyzed using SAS version 9.3 (Cary, NC) to provide descriptive statistics and correlation coefficients to compare the data collected using the MDT with the data collected by the independent observers. A Bland-Altman plot was constructed to compare the correlation between Birth and Cry and Spearman correlations to compare time between Birth and BMV.

Descriptive statistics were used to present the results from the convenience sample of BAs that participated in a survey about their experiences using the MDT.

The study was approved by the ethics committees under the JNMC Institutional Ethics Committee on Human Subjects Research (IORG#0001102) and under the RTI International Institutional Review Board amendment (ID # 12940).

## Results

The validation study began May 15, 2013 and data were collected through October 31, 2013 in the study health facilities. During this period, 2,107 (46 %) deliveries of the total of 4,597 deliveries during the study period, were timed with MDT (Table 1). 1,960 (95 %) neonates breathed within 1 min of delivery; 44 (2 %) and 3 (0.1 %) of them required 1–2 min and >2 min, respectively, to begin breathing. Only 41 (2 %) of the babies required BMV. Because of technical problems with the recordings, the time to breathing was not confirmed for 52 (2 %) of those timed.

**Table 1:** Mobile Device Timer (MDT) deliveries in five HBB trained facilities

	GH Ramadurg	KHI	CHC Sankeshwar	GH Gokak	GH Chikkodi	Total
	N (%)	N (%)	N (%)	N (%)	N (%)	N (%)
Number of total deliveries						4,597
Number of deliveries timed	493	441	271	441	461	2,107(46)
Infant status						
Alive	489 (99.2)	441 (100.0)	270 (99.6)	440 (99.8)	461 (100.0)	2,101 (99.7)
Macerated stillbirth	4 (0.8)	0 (0.0)	1 (0.4)	1 (0.2)	0 (0.0)	6 (0.3)
Baby cried^a^						
Yes	488 (99.8)	424 (96.1)	265 (98.1)	436 (99.1)	446 (96.7)	2,059 (98.0)
No	1 (0.2)	17 (3.9)	5 (1.9)	4 (0.9)	15 (3.3)	42 (2.0)
Baby ventilated^a^						
Yes	1 (0.2)	16 (3.6)	5 (1.9)	4 (0.9)	15 (3.3)	41 (2.0)
No	0 (0.0)	1 (0.2)	0 (0.0)	0 (0.0)	0 (0.0)	1 (0.0)
NA^b^	488 (99.8)	424 (96.1)	265 (98.1)	436 (99.1)	446 (96.7)	2,059 (98.0)
Time between birth and cry ^a^						
≤1 min	458 (93.9)	395 (93.2)	245 (92.5)	424 (97.2)	438 (98.2)	1,960 (95.2)
1–2 min	7 (1.4)	17 (4.0)	14 (5.3)	2 (0.5)	4 (0.9)	44 (2.1)
>2 min	1 (0.2)	0 (0.0)	1 (0.4)	0 (0.0)	1 (0.2)	3 (0.1)
<0, edit pending	2 (0.4)	0 (0.0)	2 (0.8)	0 (0.0)	0 (0.0)	4 (0.2)
Recording problem	20 (4.1)	12 (2.8)	3 (1.1)	10 (2.3)	3 (0.7)	48 (2.3)
Time between birth and bag and mask ventilation^a^	1	17	5	4	15	42
≤1 min	1 (100.0)	6 (35.3)	0 (0.0)	2 (50.0)	10 (66.7)	19 (45.2)
1–2 min	0 (0.0)	10 (58.8)	5 (100.0)	2 (50.0)	4 (26.7)	21 (50.0)
>2 min	0 (0.0)	1 (5.9)	0 (0.0)	0 (0.0)	1 (6.7)	2 (4.8)

^a^ Excludes macerated stillbirths ^b^ cried immediately after birth

**Fig. 6:**
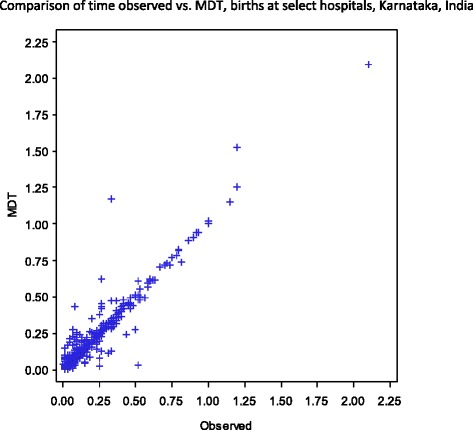
Comparing Time between Birth and Cry for Observed and MDT Data. Comparison of time observed vs. MDT, births at select hospitals, Karnataka, India

**Fig. 7:**
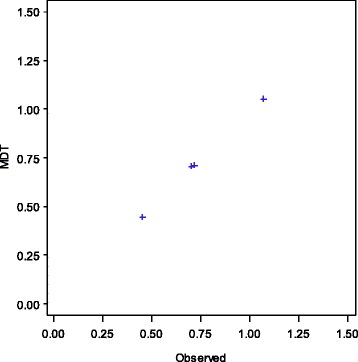
Comparing time between Birth and Ventilation for Observed and MDT Data

**Fig. 8:**
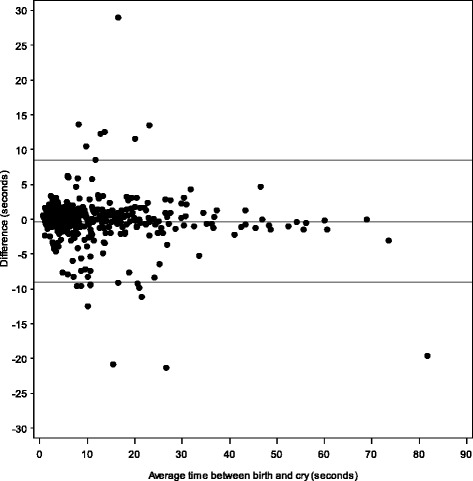
Bland-Altman Plot of Time Between Birth and Cry for Observed and MDT Data (Exclude 2 Outliers)

Trained nurses observed 438 deliveries, 21 % of the total 2,107 deliveries timed with MDT (Table 2). There was a significant positive correlation between the MDT time interval data and the observed data when comparing the time between Birth and Cry (r = .9488, *p* = 0.0005) (Fig. 6) and the time between birth and BMV (r = 1.00, *p* = 0.0005) (Fig. 7). A Bland-Altman plot illustrates the time between birth and cry for the observed and MDT data (Fig. 8).

**Table 2:** Comparison between observed and MDT deliveries

	Observer data	MDT data	Correlation	*P*-value
	N (%)	N (%)		
Number of MDT deliveries that were observed	438	438		
Time between birth and cry*	434	434	0.95	<.0001
≤1 min	430 (99.1)	427 (98.4)		
1–2 min	3 (0.7)	6 (1.4)		
>2 min	1 (0.2)	1 (0.2)		
Time between birth and ventilation*	4	4	1.00	<.0001
≤1 min	3 (75.0)	3 (75.0)		
1–2 min	1 (25.0)	1 (25.0)		
>2 min	0 (0.0)	0 (0.0)		

*p Value < .0001

### Feedback from the BAs

Of the 46 active BAs among the five facilities, 30 participated in a written survey about their experiences with the use of the MDT. Nineteen (63 %) of them had used Android phones prior to their participation in this study. More than three-quarters (76 %) of them were satisfied with the training on the use of MDT. Twenty-one (70 %) and 4 (13 %) of the BAs felt that the application was very beneficial and extremely beneficial, respectively, to reflect on their performance and in achieving Golden Minute resuscitation in subsequent events. Four-point Likert’s Scale (Table 3) were used to collect responses on the ease/difficulty of using the MDT, including starting the device, obtaining quality voice records, filling out the information on the mobile phone and synchronization.

**Table 3:** Responses on the ease of using the MDT

Item	Extremely difficult (Not user friendly)	Slightly difficult to use	Moderately easy to use	Extremely easy to use
How easy it was to use this Android based application in terms of starting the timer device?	0 (0 %)	4 (13.3 %)	12 (40.0 %)	14 (46.6 %)
How easy it was to use this Android based application in terms of getting quality voice records for events (Birth and Cry/Bag)?	0 (0 %)	5 (16.7 %)	8 (26.7 %)	17 (56.7 %)
How easy it was to use this Android based application in terms of filling out the information on Mobile phone?	0 (0 %)	3 (10.0 %)	8 (26.7 %)	19 (63.3 %)
How easy it was to use this Android based application in terms of synchronization/data transfer?	0 (0 %)	3 (10.0 %)	6 (20.0 %)	10 (33.3 %)

## Discussion

We demonstrated the feasibility of using an application on a MDT to time delivery room events. This simple MDT provided valid observations of the time to crying/spontaneous respiration or BMV and was feasible to use in the low-resource setting. This is the first Android application, to our knowledge, to facilitate automatic timing of significant birth events. The BAs were able to use the devices successfully with minimal training and the recordings provided valid event timing. The majority of the BAs (83 %) felt that MDT helped them to ensure that successful ventilation was achieved within the Golden Minute. This type of device may be useful in reinforcing HBB and similar newborn resuscitation training.

This pilot study was designed to test the feasibility and acceptability of using an Android type system to collect small amounts of data. This was a pragmatic study aimed at capturing a convenience sample of deliveries thus one limitation of the study was that we were able to time less than 50 % of the deliveries with MDT due to a variety of reasons. Of the 124 BAs trained for HBB in the five facilities, only 46 BAs actually used the MDT. Therefore, only births conducted during the time the participating BAs were present in the delivery room were timed. We initially trained five facility coordinators who in turn trained other BAs working in their facilities. Hence, the training for the use of MDT occurred over a period of time and consequently not all births could be timed. Caesarean deliveries (*n* = 690) and those who arrived to the delivery room with signs of imminent delivery were excluded. While the Android application was useful in the field testing, the survey results suggested that further refinement and assessment using sound/voice recognition instead of the manual process to determine the time difference. Further research is needed to confirm its usefulness for a wider application. In addition, this study had limitations regarding the small number of babies requiring BMV; however, with the increasing emphasis on stimulation in HBB training, this result is not altogether surprising [4–6, 12–14]. This was a pilot study aimed at whether or not the BAs would use the MDT device. A larger study in less controlled environments may be required to assess the feasibility and larger applicability of the device. In essence, the primary focus of the MDT is to assess the feasibility of Golden Minute resuscitation by BAs trained for HBB in diverse delivery settings. Feasibility of timely resuscitation is a question that needs to be addressed, especially in resource limited settings with a single birth attendant caring for the mother and the newborn. This is not a limitation of this MDT validation study, but, rather of the HBB training program itself and the MDT may well help us answer that question in a more objective manner.

## Conclusions

The goal of any resuscitation effort is to establish spontaneous respiration at the earliest and prevent insult from to the newborn. The MDT is a tool for the BAs to evaluate their proficiency in achieving it and improve on performance with each resuscitation experience. Simple, effective tools such as this are needed to reinforce safe delivery practices, especially in low-resource settings. With improvement, this type of device may be useful in international efforts to improve newborn resuscitation, both for research and clinical practice.

## Abbreviations

HBB: Helping babies breathe
AAP: American academy of pediatrics
BA: Birth attendant
NICHD: National institute of child health and human development
BMV: Bag and mask ventilation
MDT: Mobile delivery-room timer
CHC: Community health center
